# Inaccurate viral prediction leads to overestimated diversity of the archaeal virome in the human gut

**DOI:** 10.1038/s41467-024-49902-w

**Published:** 2024-07-17

**Authors:** Cynthia M. Chibani, Shiraz A. Shah, Ruth A. Schmitz, Stephen Nayfach

**Affiliations:** 1 Institut für Allgemeine Mikrobiologie, Christian-Albrechts-Universität zu Kiel, Am Botanischen Garten 1–9, D-24118 Kiel, Germany; 2 Copenhagen Prospective Studies on Asthma in Childhood, Copenhagen University Hospital, Herlev-Gentofte, Gentofte Denmark; 3 Profluent Bio, Berkeley, CA USA; 4 Joint Genome Institute, Berkeley, CA USA

**Keywords:** Computational biology and bioinformatics, Microbiology, Metagenomics

**arising from** R. Li et al. *Nature Communications* 10.1038/s41467-022-35735-y (2022)

Inclusion of curated archaeal virus genomes in public databases is a critical step towards uncovering the distribution and evolution of archaeal viruses in the microbiome^1^. In a recent study, Li et al.^2^, created the Human Gut Archaeal Virome Database (HGAVD), which is claimed to comprise genomes of 1279 species of archaeal viruses, representing a >13-fold increase in archaeal virus diversity compared to previous studies^3–5^. However, re-analysis of the HGAVD revealed extensive contamination from Bacteria and Archaea, with 72–83% of sequences classified as non-viral by six different viral prediction tools. An improved reference database of archaeal genomes is needed to avoid propagation of errors in future studies and to accurately characterize the role of the archaeal viruses in the microbiome.

Intrigued by the large expansion in archaeal viral diversity relative to recent studies, we used six state-of-the-art computational tools, including CheckV v1.0.1^6^, geNomad v1.5.0^7^, VIBRANT v1.2.1^8^, ViralVerify v1.1^9^, VirSorter v1.0.6^10^, and lastly VirSorter2 v2.2.4^11^ using default parameters to perform post-hoc analysis on the HGAVD, demonstrating it was composed primarily of **non-viral sequences** (Fig. 1A, B and Supplementary Data 1). Of the 1279 sequences in the HGAVD, only 30.88% were predicted as a virus or provirus by *any* of the six tools and only 14.46% by all six. While archaeal viruses may be more challenging to detect in microbiome samples^1^, nearly all non-viral HGAVD sequences (985 of 987) were confidently assigned as either Archaea or Bacteria by geNomad as opposed to other mobile genetic elements. Viral classification can be challenging for very short sequences, but even long HGAVD sequences (>10 kbp) were found to contain tens to hundreds of host-specific genes while lacking any virus-specific gene (Fig. 1C). Together, the computational tools we used sensitively classified 91 of 92 archaeal viruses from NCBI RefSeq as viral, indicating our result is not a byproduct of false negatives (Supplementary Data 2).

**Fig. 1:**
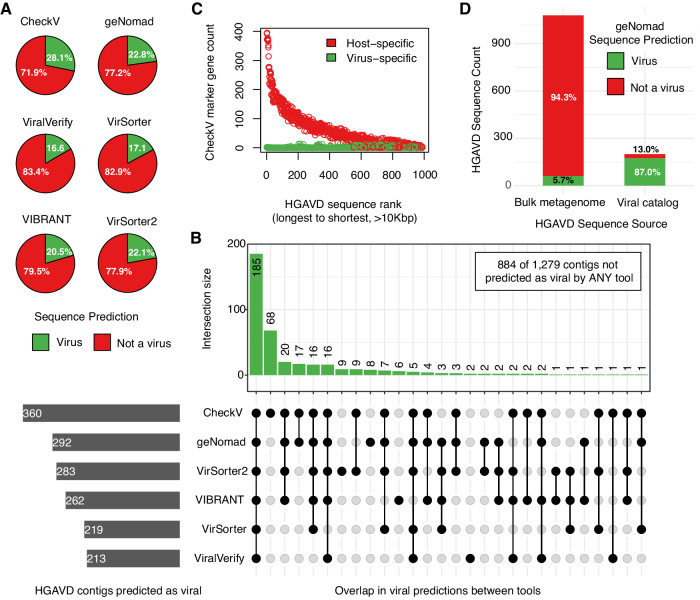
Analysis of 1279 putative archaeal viruses from the HGAVD. **A** The prediction result of six different viral classifiers for the HGAVD. Predicted proviruses were counted as viruses. **B** Upset plot representing the number of shared/unique viral predictions across the six tools. **C** Counts of virus-specific and host-specific proteins identified on 989 HGAVD contigs longer than 10 kbp. Sequences are sorted from longest to shortest. The longest HGAVD sequences have numerous host-specific genes and few viral genes. **D** geNomad viral predictions for HGAVD contigs based on data source. Most false positives originate from bulk metagenomes not included in previously published viral genome catalogs.

Next, we searched for the source of the prediction error. To identify viruses, Li et al. used a combination of sequence matches to putative viral signature genes and sequence matches to archaeal CRISPR spacers. Most of the Li et al. signature genes matched two other viral databases (VOGDB http://vogdb.org/ and VPF^12^ confirming their viral origin, and most HGAVD sequences contained matches to Li et al. signature genes. However, only 27.36% of HGAVD sequences contained matches to *virus-specific* genes from three curated databases (CheckV, geNomad, and Virsorter2), suggesting that many of the putative signature genes from Li et al. are not specific to viruses. We also confirmed that nearly all HGAVD sequences contained matches to archaeal CRISPR spacers (see Supplementary Information). It is known that CRISPR spacers sometimes target chromosomal genes that are involved in plasmid conjugation or replication^13^ and that viruses often exchange genes with their hosts^14^. Thus neither of the signals are sufficient to perform accurate virus classification. To remove non-viral sequences, Li et al. relied on alignment to genomes of gut-isolated archaea (*n* = 35) and bacteria (*n* = 10,613). However, when we aligned the HGAVD to a larger collection of 1825 archaeal genomes from RefSeq, 59.5% of HGAVD sequences contained a match with > 90% identity over >90% sequence length. Consistent with this result, we found that most of non-viral sequences in the HGAVD were identified from bulk metagenomes (containing a mixture of sequences from viruses and cellular organisms) as opposed to previously published databases of curated viral genomes (Fig. 1D).

As an illustrative example, the largest sequence in the HGAVD was 560,083 bp, which would make this the largest virus genome discovered from the human gut microbiome (553,716 bp^4^), and the largest sequenced genome of any archaeal virus (216,805 bp^15^). However, alignment against NCBI RefSeq revealed a robust match to archaeal type strain *Methanobrevibacter smithii* ATCC 35061 (99% identity over 93% of the sequence length), and visual inspection^16^ revealed numerous genes for host metabolism and cellular processes, even including 16 S rRNA (Fig. S1). While the sequence did contain CRISPR spacer matches, no prophages could be identified using geNomad or VIBRANT, and no *virus-specific* genes were identified by either geNomad or Virsorter2.

Together, our analyses clearly demonstrate that the sequences reported by Li et al. are highly contaminated by cellular organisms and should not be utilized as a reference database for viral analyses. A more careful and systematic analysis is needed to accurately characterize the diversity of archaeal viruses in the human gastrointestinal tract and establish a high-quality reference collection. While novel approaches for viral detection can yield new discoveries, they should be carefully benchmarked in terms of sensitivity and specificity. In the absence of such benchmarking, we recommend using well-established virus detection tools, like geNomad or VirSorter2, which can distinguish sequences of viruses from cellular organisms and other mobile genetic elements^12^.

## Reporting summary

Further information on research design is available in the Nature Portfolio Reporting Summary linked to this article.

### Supplementary information

Supplementary information

Description of Additional Supplementary Files

Supplementary Data 1

Supplementary Data 2

Reporting Summary

## Data Availability

Datasets generated and/or analyzed during the current study are available as supplementary data.

## Code availability

Code to reproduce the analyses in this paper is available at: https://github.com/CynthiaChibani/HGUT_arch_viruses.
