# Supplementary material for: Inaccurate viral prediction leads to overestimated diversity of the archaeal virome in the human gut
Source: Nat Commun. 2024 Jul 17;15:5976. doi: 10.1038/s41467-024-49902-w (PMC11255274; doi:10.1038/s41467-024-49902-w)
Supplement: Supplementary file 1 — Supplementary information [file 41467_2024_49902_MOESM1_ESM.docx]

# Supplementary Figures

#
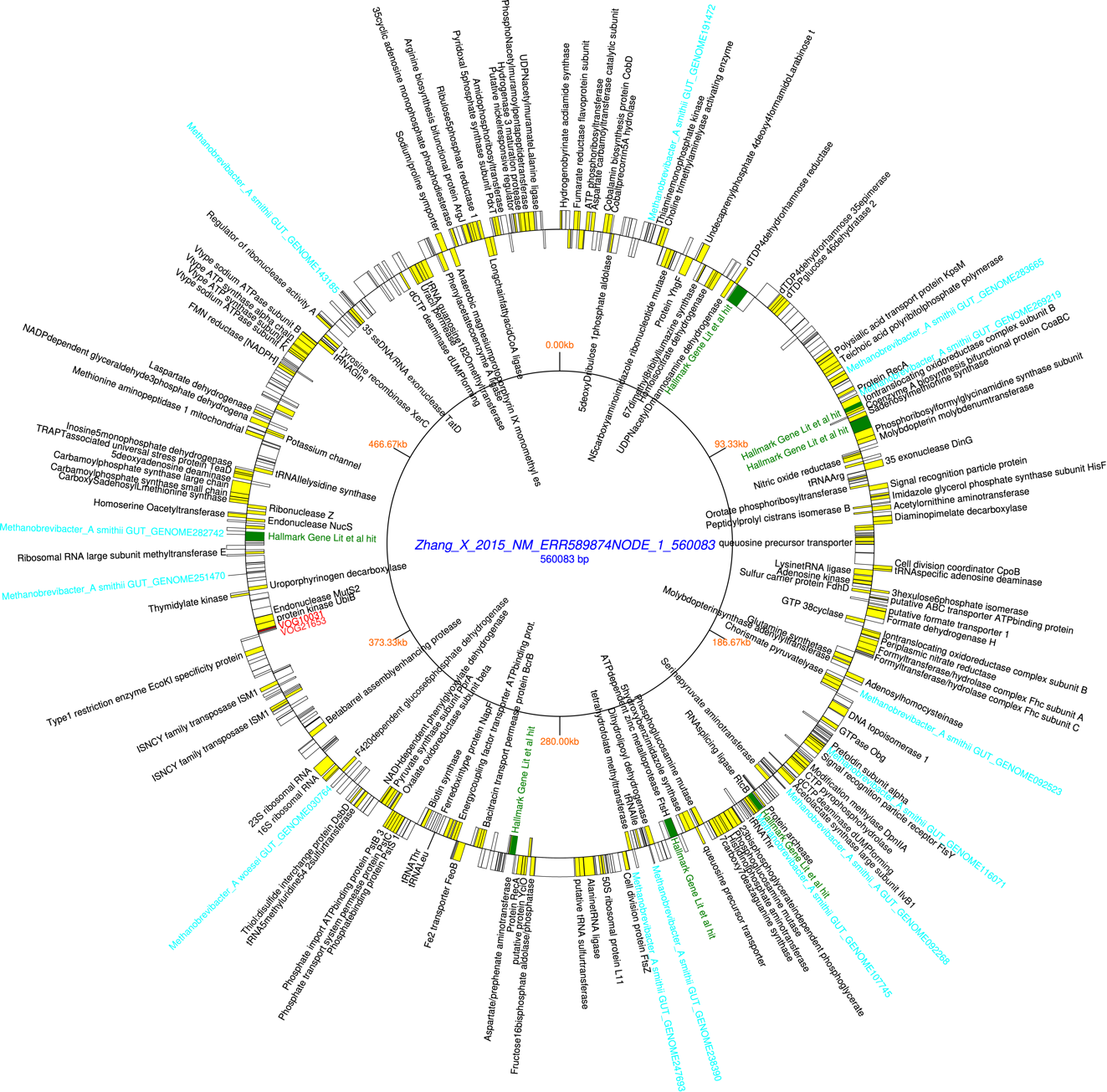


# **Figure. S1**. Genome map of the largest sequence of 560 kb of the HGAVD. Hypothetical genes are colored in white, predicted ORFs are colored in yellow, viral genes are highlighted in red, and nucleotide sequences containing CRISPR spacer matches are highlighted in light blue.

# Supplementary Information

## Virus prediction software

HGAVD sequences were downloaded from Figshare (<https://doi.org/10.6084/m9.figshare.21152404.v3>) and analyzed with six viral prediction tools using default parameters: VirSorter v1.0.6 (Roux et al., 2015), VirSorter2 v2.2.4 (Guo et al., 2021), VIBRANT v1.2.1 (Kieft et al., 2020), ViralVerify v1.1 (Antipov et al., 2020), geNomad v1.5.0 (Camargo et al., 2023), and CheckV v1.0.1 (Nayfach et al., 2021b). VirSorter identified 219 viral sequences (categories 1 and 2). VirSorter2 identified 283 viral sequences with at least hits to one viral hallmark gene as our selected cut-off criteria. VIBRANT identified 262 viral sequences, including 11 that were predicted as integrated proviruses. All contigs predicted by VIBRANT as lytic or lysogenic were selected as viruses for this analysis. ViralVerify identified 213 viral sequences, 5 plasmids, and 681 chromosomal sequences, with the remaining 380 sequences flagged as uncertain. GeNomad v1.5.0 identified 292 viruses (including 37 proviruses) and 2 plasmids. Lastly, CheckV v1.0.1 identified 360 viruses where contigs with at least one hit to a viral-specific gene. Note: CheckV was not originally developed as a virus prediction tool and our chosen threshold is likely too permissive in practice.

We used the same six viral identification tools and the same selection criteria to classify 92 archaeal viruses from NCBI RefSeq (date of access 03/07/2023; Table S2). VirSorter identified 40 viral sequences (categories 1 and 2), VirSorter2 identified 71 viral sequences with at least hits to one viral hallmark gene, VIBRANT identified 87 archaea viruses, ViralVerify identified 29 viral sequences, geNomad identified 86 viruses, and lastly, CheckV identified 74 viruses with at least one hit to one viral hallmark gene. Taken together, 91 out of 92 archaeal viral genomes from NCBI RefSeq were predicted to be viral by the six computational tools.

## Viral hallmark proteins

We evaluated the validity of putative viral hallmark genes identified by Li et al. First, we scanned the putative hallmarks against VOGDB (release 202; <http://vogdb.org/>), and VPF (Paez-Espino et al., 2017), two of the largest databases of profile HMMs for virus-specific proteins. We used the hmmsearch command in HMMER3 (e-value cutoff 1e-5) (Eddy et al., 2011) to search 8,485 proteins that were selected as the hallmark genes for archaeal viruses against the VOGDB which resulted in 6,673 hits, and against the VPF which resulted in 3,461 hits. A total of 7,230 out of the 8,485 putative hallmark genes (85.25%) had hits to either VPF or VOGDB. Next, we functionally annotated the hallmark genes using EggNOG mapper v2.0 (Cantalapiedra et al., 2021) and the eggNOG database v.5.0 (Huerta-Cepas et al., 2019). Several genes contained annotations with virus-specific keywords like portal (n=15), terminase (n=44), capsid (n=5), sheath (n=2), tail (n=23), virion (n=2), baseplate (n=14), and head (n=9). This result indicates that the hallmark genes selected by Li et al. mostly overlap with publicly available viral genes. Lastly, we evaluated the number of HGAVD sequences that contained a viral hallmark gene. We reproduced the method used by Li et al. using DIAMOND (e-value cutoff 1e-5) (Buchfink et al., 2021) to perform a translated alignment of HGAVD nucleotide sequences against the 8,485 hallmark proteins.

## Viral spacer matches

Besides viral hallmarks, the authors constructed and used the Human Gut Associated Archaeal Spacer Database (HGASDB) for archaeal virus identification. To recapitulate the analysis from Li et al. we used the CRISPR Recognition Tool v1.1 (CRT) with default parameters (Bland et. al, 2007) to identify CRISPR spacers from 1,162 archaeal MAGs and isolate genomes plus an additional five archaeal MAGs we previously identified (Chibani et al., 2022). BLASTn was used to identify exact matches and matches with one base difference between the spacer collection and the HGAVD. Out of the 1,279 sequences of the HGAVD, 1,204 sequences had hits to spacers predicted on the archaeal genomes (94.1%).

Archaeal genome alignment

HGAVD sequences were aligned to 1,825 archaeal genome assemblies from RefSeq (release 220, downloaded on Nov 11, 2023). BLASTn v2.14.1+ was run using options: -outfmt '6 std qlen slen' -max_target_seqs 10000. BLASTn output file was used to calculate nucleotide identity and alignment coverage between each HGAVD sequences and RefSeq contig using the script: https://bitbucket.org/berkeleylab/checkv/src/master/scripts/anicalc.py.

# References

1. Buchfink, B., Reuter, K. and Drost, H.G., 2021. Sensitive protein alignments at tree-of-life scale using DIAMOND. Nature methods, 18(4), pp.366-368.
2. Huerta-Cepas, J., Szklarczyk, D., Heller, D., Hernández-Plaza, A., Forslund, S.K., Cook, H., Mende, D.R., Letunic, I., Rattei, T., Jensen, L.J. and von Mering, C., 2019. eggNOG 5.0: a hierarchical, functionally and phylogenetically annotated orthology resource based on 5090 organisms and 2502 viruses. Nucleic acids research, 47(D1), pp.D309-D314.
3. Cantalapiedra, C.P., Hernández-Plaza, A., Letunic, I., Bork, P. and Huerta-Cepas, J., 2021. eggNOG-mapper v2: functional annotation, orthology assignments, and domain prediction at the metagenomic scale. Molecular biology and evolution, 38(12), pp.5825-5829.
4. Eddy, S.R., 2011. Accelerated profile HMM searches. PLoS computational biology, 7(10), p.e1002195.
