# Supplementary material for: Inaccurate viral prediction leads to overestimated diversity of the archaeal virome in the human gut
Source: Nat Commun. 2024 Jul 17;15:5976. doi: 10.1038/s41467-024-49902-w (PMC11255274; doi:10.1038/s41467-024-49902-w)
Supplement: Supplementary file 2 — Description of Additional Supplementary Files [file 41467_2024_49902_MOESM2_ESM.pdf]

File Name: Supplementary Data 1
Description: Output of six viral prediction tools for 1,279 HGAVD sequences

File Name: Supplementary Data 2
Description: Output of six viral prediction tools for 92 archaeal viruses from NCBI RefSeq
